# Imaging Meets Cytometry: Analyzing Heterogeneous Functional Microscopic Data from Living Cell Populations

**DOI:** 10.3390/jimaging7010009

**Published:** 2021-01-13

**Authors:** Matthew Draper, Mara Willems, Reshwan K. Malahe, Alexander Hamilton, Andrei I. Tarasov

**Affiliations:** 1 School of Biomedical Sciences, Ulster University, Cromore Road, Coleraine BT52 1SA, UK; mbdraper123@live.co.uk; 2 Oxford Centre for Diabetes, Endocrinology and Metabolism, University of Oxford, Churchill Hospital, Headington, Oxford OX3 7LE, UK; marawillems@gmail.com (M.W.); s.r.k.malahe@umail.leidenuniv.nl (R.K.M.); alexander.hamilton@med.lu.se (A.H.); 3 Lund University Diabetes Centre, Unit of Molecular Metabolism, Clinical Research Centre, Malmö University Hospital, 20502 Malmö, Sweden; 4 Oxford Biomedical Research Centre, National Institute for Health Research, Oxford OX3 7LE, UK

**Keywords:** time-lapse imaging, fluorescence, cell profiling, baseline correction, antidiabetic drugs

## Abstract

Biological tissue consists of populations of cells exhibiting different responses to pharmacological stimuli. To probe the heterogeneity of cell function, we propose a multiplexed approach based on real‐time imaging of the secondary messenger levels within each cell of the tissue, followed by extraction of the changes of single‐cell fluorescence over time. By utilizing a piecewise baseline correction, we were able to quantify the effects of multiple pharmacological stimuli added and removed sequentially to pancreatic islets of Langerhans, thereby performing a deep functional profiling for each cell within the islet. Cluster analysis based on the functional profile demonstrated dose‐dependent changes in statistical inter‐relationships between islet cell populations. We therefore believe that the functional cytometric approach can be used for routine quantitative profiling of the tissue for drug screening or pathological testing.

## 1. Introduction

Biological tissue is an ensemble of cells implementing a specific common function. These are frequently cells of different types, each type running a different “subroutine” of the main functional program. An example of this arrangement is pancreatic islet of Langerhans, in which cells secreting a glucose-lowering hormone insulin (β-cells) are positioned next to their counter-parts secreting glucagon (α-cells) that, in turn, elevates blood glucose [1]. The unwritten biological convention is that all the cells of the same type are *roughly* alike. For instance, it is believed that the mutations in an energy-sensing molecule (the so-called ATP-sensitive K^+^ (K_ATP_) [2,3,4] channel) render *roughly all* pancreatic islet β-cells incapable of sensing blood glucose. At the same time, adrenaline is believed to induce secretion of glucagon from *roughly all* α-cells [5,6], to rescue the body from hypoglycemia.

Whilst valid in a broad sense, this assumption, however, has its limits, as different cells of the same type are not absolutely identical. This phenotypical heterogeneity may stem from an exposure to different factors of microenvironment [7], such as neighboring cells [8], local signals [9] or biological polarity [10]. Secondly, biological responses have stochastic molecular nature, which makes them subject to variability. For the examples above, energy sensing by the K_ATP_ channel is based on its ability to bind the molecule of ATP [11,12], whilst the α-cell sensitivity to adrenaline stems from how densely the β-adrenergic receptors are expressed on its membrane.

The heterogeneity of cell phenotype can be probed by flow cytometry, a high-throughput technology that reports the expression of several proteins on the membrane of the same cell at once, by staining the cell suspension with monoclonal antibodies conjugated with fluorescent markers. Deep phenotypical profiling of living cells, arranged into an intact tissue, is done via high-content microscopy [13,14], which also characterizes specific proteins expressed on the plasma membrane. An obvious shortcoming of these otherwise powerful profiling techniques is their reliance on cell structure, which is not equivalent to the true function of the living cells. A more function-oriented approach, real-time imaging of the intracellular levels of secondary messenger compounds, such as cAMP or Ca^2+^, with fluorescent sensors, can profile the subpopulations of cells via specific pharmacological stimuli [5,15,16], the so-called “marker compounds” [17]. The real-time imaging, however, cannot match the statistical power of the approaches above, due to a lower throughput and an increased experimental duration. Imaging of a response to a single specific stimulus may take tens of minutes, and the responses to stimuli added sequentially are difficult to interpret due time-dependent drift of fluorescence. 

In this paper, we aimed to develop an experimental and analytical framework for deep profiling of large populations of intact living cells based on differential response to multiple pharmacological compound(s). We specifically focus on pancreatic islets of Langerhans as a model and image the cytosolic level of the secondary messenger cAMP, as this compound (i) changes on a slow (minutes) timescale, which is advantageous for imaging of large cell populations, and (ii) cannot be transmitted between two neighboring cells, unlike cytosolic Ca^2+^ or electrical potential of plasma membrane.

Importantly, cAMP signaling in pancreatic islet cells is a target of physiological regulation by incretins, natural highly selective insulinotropic peptide agents secreted by the gut cells. Glucagon-like peptide-1 (GLP-1), secreted by the gut L-cells; enhances the secretion of insulin and attenuates secretion of glucagon, in a glucose-dependent manner [18]. This mode of action makes GLP-1 an excellent antidiabetic medication as per se it is not able to induce hypoglycemia, a major problem with many antidiabetic drugs. The active form of GLP-1 (known as “7–36” form) has a short lifetime in circulation as it is rapidly inactivated by a ubiquitous enzyme, dipeptidyl peptidase-4 (DPP-4), to the inactive “9–36” form. Gastric inhibitory peptide (GIP), secreted by the gut K-cells effectively works in a way similar to that of GLP-1’s, inclusive of being inactivated by DPP-4 [19]. The two key differences between the GLP-1 and GIP action are (i) the lack of inhibition of glucagon secretion and (ii) less reliable and therefore yet unexplored therapeutic perspectives of the latter, as exogenous GIP has only a small effect in human patients [19].

Critical steps in imaging of intracellular concentration of cAMP ([cAMP]_i_) were achieved upon development of a fluorescent genetically encoded sensors able to report submicromollar changes in [cAMP] [20] as well as the activity of proteins kinase A (PKA) [21], a parameter that is directly dependent on [cAMP]_i_. We utilized the PKA and cAMP sensors for deep multifactor (ca.10 agonists) profiling of 100 s–10,000 s of cells obtained throughout long (multihour) recording periods on an automatic imaging system.

## 2. Materials and Methods

### 2.1. Animals and Islet Isolation

All mouse experiments were conducted in accordance with the United Kingdom Animals (Scientific Procedures) Act (1986) and the University of Oxford ethical guidelines. C57Bl6 mice (Charles River, Harlow, UK) were killed by cervical dislocation and pancreatic islets were isolated as detailed in [17]. Islets were cultured in RPMI medium containing 11 mM glucose, supplemented with 10% fetal bovine serum, 100 IU/mL penicillin and 100 μg/mL streptomycin (all reagents from Life Technologies, Paisley, UK) in absolute humidity in the atmosphere with 5% CO_2_. The recombinant sensors of [cAMP]_i_ and protein kinase A (PKA) activity were delivered using adenoviral vectors at 10^5^ infectious units per islet [22], followed by 24–36 h culturing (as above) required for the expression of the reporter proteins.

### 2.2. Imaging

**Figure 1:**
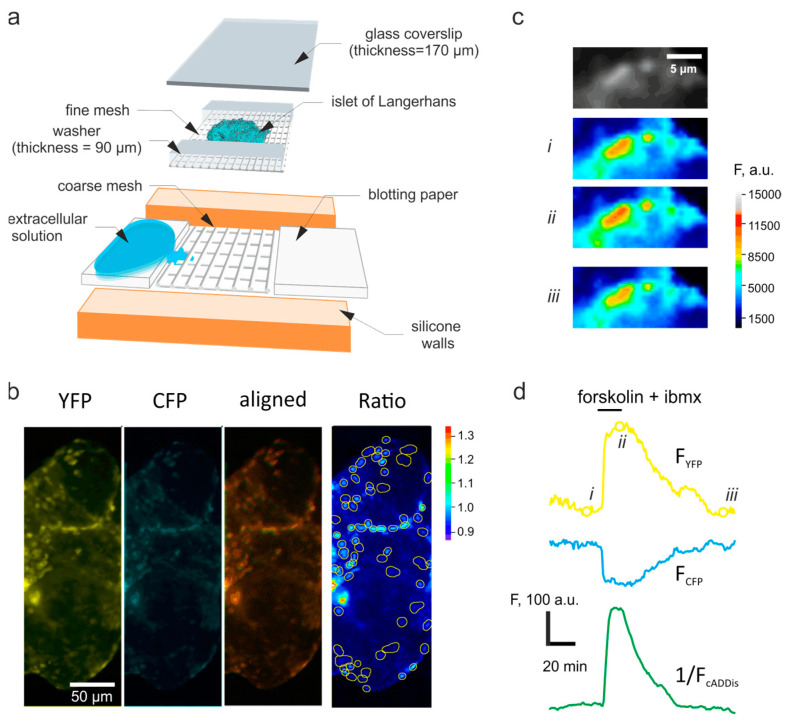
Imaging set-up, sensor expression and the FRET channels. (**a**) Schematic of the imaging chamber. (**b**) Expression of the recombinant proteins kinase A (PKA) sensor, AKAR3, in islets of Langerhans. Raw YFP, CFP channel fluorescence, imaged using epifluorescence microscopy, alignment of YFP and CFP channels, and the YFP/CFP ratio, as indicated. The individual cells (regions of interest) detected as intensity maxima, are outlined in yellow on the ratio image. (**c**,**d**) Representative changes in the raw fluorescence intensity of the YFP channel (**c**) corresponding to the three indicated time-points of the raw kinetics traces (**d**) of the YFP (top, yellow), CFP (middle cyan) channels recorded pseudosimultaneously with the fluorescence of Green Downward cADDis (bottom, green).

Imaging experiments were performed using the chamber [23], modified as reported [24]. Groups of islets were loaded on the nylon mesh inside the silicone basin of the chamber using an automatic pipette, and immobilized by a gentle pressure from above, using a 24 mm× 40 mm× 0.17 mm glass coverslip (Menzel Gläser, Fisher Scientific, Loughborough, UK) (Figure 1a). The sides of the coverslip were dry-sealed to the silicone of the chamber by applying a gentle pressure. Immobilized within the chamber, the islets were perifused with the bath solution at 50 μL/min, using a peristaltic pump. The bath solution contained, mM: 140 NaCl, 4.6 KCl, 2.6 CaCl_2_, 1.2 MgCl_2_, 1 NaH_2_PO_4_, 5 NaHCO_3_, 10 glucose, 10 HEPES (pH 7.4, with NaOH) as well as the pharmacological agents, as indicated. The addition/removal of the drugs was recorded as a timestamps vector during the experiment. The imaging chamber was positioned within the temperature-controlled stage (+34 °C) of a wide-field Axiozoom.V16 microscope (Carl Zeiss, Cambridge, UK).

The signal in pancreatic islet cells was reported using recombinant probe AKAR3, a fusion of a molecule of cyan fluorescent protein (CFP), a modified variant of yellow fluorescent protein (YFP), “Venus”, covalently linked by a PKA-sensitive forkhead-associated domain (FHA) [21]. Upon excitation at 430/24 nm, the CFP domain emits at 470/24 nm, as was detected using the respective filter set. An increase in the PKA activity changes the FHA conformation, which, in turn, brings the CFP and YFP domains of the sensor in close proximity. In this case, the energy of the excited CFP domain excites the YFP domain via the Förster resonance energy transfer (FRET), which results in YFP emission detected at 535/30 nm [25]. Importantly, YFP is practically not excited at the CFP excitation wavelength, 430/24 nm [26]. Time-lapse imaging of [cAMP]_i_ was performed using recombinant Green Upward cADDis sensor (Montana Molecular, Bozeman, MT, USA) using the same microscopic system. The cADDis represents a single-fluorophore system that increases its fluorescence intensity in response to the increases in [cAMP]_i_. The cADDis fluorescence was excited at 470/40 nm and the emission recorded at 525/50 nm. PKA and cAMP were imaged in the islets every 60 s (16 mHz).

### 2.3. Image Analysis

Image sequences were analyzed (registration, background subtraction, intensity vs. time analysis) using open-source FIJI software (http://fiji.sc/Fiji, Version 1.53). The alignment of the two emission channels, corresponding to CFP and YFP, respectively, was adjusted off-line, using Cairn Image Splitter plug-in to FIJI [27] (Cairn Research, Faversham, UK) (Figure 1b). Individual cells were detected in the fluorescence images as intensity maxima of the YFP channel, as detailed in [28]. Briefly, average intensity projection of the recorded YFP time-lapse was used as a pattern image for detection of the regions of interest (ROI):

(1) $$\bar{I}{\left(x,y\right)}_{t}=\;\frac{\sum_{t=1}^{N}I\left(x,y,t\right)}{N}$$

where I(x,y,t) is the intensity a pixel of the original 3-D image stack, with the linear coordinates x and y, *N* is the stack size (total number of time-points), *t* is the time and $\bar{I}{\left(x,y\right)}_{t}$ is the intensity of a the respective pixel of the transformed 2D image.

The intensity maxima were detected [29] by (i) detecting the local maxima of the image, based on the selected threshold intensity difference between the local pixels, $I_{prom}$, and (ii) performing a flood fill around each identified maxima based on a tolerance threshold, $k_{tol}$. The separate thresholded areas were recognized as regions of interest (ROI) [30], the objects of linear sizes > 30 μm having been excluded as artefacts. The mean grayscale intensity $\bar{I}\left(c_{i},t_{j}\right)$ was then calculated for each of the *m* identified ROI (*c_i_*) within each of the *n* timeframes (*t_j_*) of the image, separately for the CFP and YFP channel images, resulting in a 2D intensity vs. time matrix:

(2) $$I_{ivt}=\left(\begin{array}{ccc} \bar{I}\left(c_{1},t_{1}\right) & \ldots & \bar{I}\left(c_{m},t_{1}\right)\\ \ldots & \bar{I}\left(c_{i},t_{j}\right) & \ldots \\ \bar{I}\left(c_{1},t_{n}\right) & \ldots & \bar{I}\left(c_{m},t_{n}\right) \end{array}\right)$$

### 2.4. Time-Lapse Data Analysis

**Figure 2:**
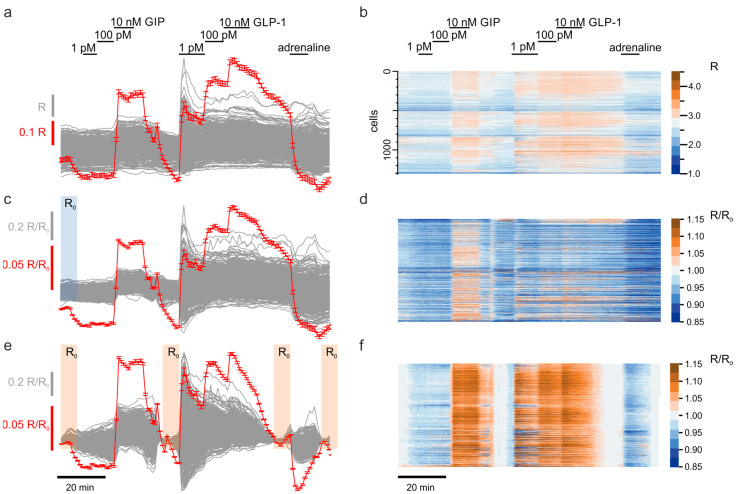
Baseline correction for populational time-lapse images. Changes in the cytosolic [cAMP] visualized via activity of PKA in response to various (ant)agonists, as indicated, recorded from a population of pancreatic islet cells. (**a**) raw data (grey traces, grey bar) and the average ± SEM trace (red). (**b**) As in (**a**) but color-coded according to the scale provided to show the response from each individual cell. (**c**) Raw data (grey) normalized to the initial fluorescence (*R/R*_0_), as indicated, average ± SEM (red). (**d**) Color-coded heat map of the data in (**c**). (**e**) Baseline-corrected data, using the baseline reference areas indicated. (**f**) Color-coded data from (**e**).

The numerical data ($I_{ivt}^{\left(CFP\right)},I_{ivt}^{\left(YFP\right)}$) was then analyzed using IgorPro package (Wavemetrics) [28]. The raw data was expressed as the per-element ratio (*R*) of the intensity of the YFP to CFP channels (Figure 2a):

(3) $$R=I_{ivt}^{\left(YFP\right)}/I_{ivt}^{\left(CFP\right)}$$

To account for the variations caused by the differences in the expression of the recombinant sensor, the data was presented as *R/R*_0_ (Figure 2b), for which each column of the 2D matrix of the intensity ratios *R* was normalized by the vector of the initial intensities for each ROI, *R*_0_, which was computed as an average fluorescence within first five time points:

(4) $$R_{0}=\left\{\overset{5}{\underset{t=1}{\sum }}R_{1,t}\ldots \overset{5}{\underset{t=1}{\sum }}R_{m,t}\right\}$$

The time-dependent drift of the sensor fluorescence was derived by recurrently imposing basal conditions, corresponding to the lack of any (ant)agonist on the experimental sample, throughout the experiment. Every return to the basal conditions (timepoints denoted below as “$t_{basal}$”) was assumed to restore the initial levels of the analyte (PKA activity or [cAMP]_i_), independently of other factors. An individual baseline trace was generated for each column of the *R/R*_0_ matrix by interpolating (Levenberg-Marquardt least-squares) the column data corresponding to multiple $t_{basal}$ and subtracted subsequently from the *R/R*_0_ data. 

We compared several interpolation functions. For linear and exponential interpolation, the first and the last of the $t_{basal}$ regions (“*R*_0_” in Figure S1a,b) were used, whereas cubic B-spline and polynomial interpolation utilized all the $t_{basal}$ regions (multiple *R*_0_*s* in Figure S1c,d). The degree of the polynomial was chosen equal to the number of the basal regions minus one. Piecewise baseline functions (Figure S1e,f) were generated using linear (Figure S1e) or square (Figure S1f) fit of each non-basal region, using the two nearest, preceding and succeeding, $t_{basal}$ regions.

To quantify the effects of various (ant)agonists on the analyte, the $t_{basal}$ regions were expanded to include 20 timepoints following the addition of the agent, each expanded region including the data corresponding to both basal and experimental conditions (Figure S2b–f). To quantify the effect for each column of the *R/R*_0_ matrix, the data within each expanded region was approximated with linear (Figure S2b), square (Figure S2c), sigmoidal (Figure S2e) or Hill (Figure S2f) functions.

**Figure 3:**
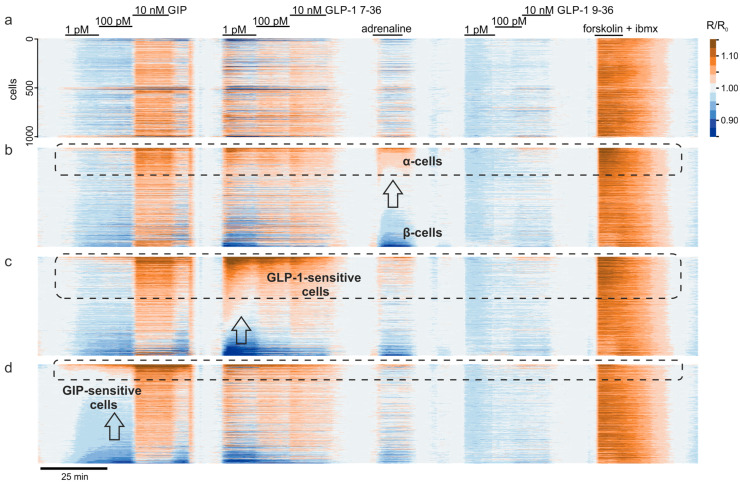
Identification of cell populations based on pharmacology. (**a**): Baseline-corrected data presented as a heat map to reflect the behavior of individual cells in response to various agonists, as indicated. (**b**): the same data but ranked according to the response to adrenaline, a marker compound for α-cells. (**c**,**d**): the same data sorted by the response to incretins GLP-1 (7–36) (**c**) and GIP (**d**).

Sorting of the columns within the *R/R*_0_ matrix (Figure 3b–d) was based on the increase of the data variance following the application of the (ant)agonist. A data range corresponding to 20 timepoints before and after (10 + 10) the application of the agonist was selected and a sorting statistic was calculated for every row of the *R/R*_0_ matrix:

(5) $$S_{s}=sgn\left(\bar{R}/{\bar{R}}_{0}-1\right)\times \sigma_{\bar{R}/{\bar{R}}_{0}}$$

where $\bar{R}/{\bar{R}}_{0}$ and $\sigma_{\bar{R}/{\bar{R}}_{0}}$ are the average *R/R*_0_ and the standard deviation of the selected range.

### 2.5. Statistics

Statistical analysis was performed using R [31]. Hierarchical clustering with the Ward’s agglomeration method was done using the *hclust* function from the core R distribution. The k-means cluster analysis was performed using the *kmeans* function of the R core and visualized using the *factoextra* library. The optimal number of clusters was calculated using the elbow method implemented in the *NbClust* library. Sample sizes are specified in the figure legends.

## 3. Results

The conditions of the adenoviral infection were optimized to allow the expression of the recombinant sensor in the predominant majority of the islet cells, whilst avoiding cell death or damage (Figure 1b). To verify the fact that we can reliably image the changes in [cAMP]_i_ and PKA activity, a conventional positive control was applied using the combination of two known agonists, forskolin (10 μM) and 3-isobutyl-1-methylxantine (IBMX, 100 μM). The two chemicals reversibly act on intracellular enzymes, adenylyl cyclase and phosphodiesterase, respectively, to elevate the cytosolic concentration of cAMP and hence activate PKA. In our hands, the application of forskolin and IBMX reversibly increased the fluorescence of the YFP channel and decreased the fluorescence of the CFP channel of the ACAR3 sensor (Figure 1c,d). The onset of the increase in the YFP/CFP ratio coincided with an increase in [cAMP]_i_ imaged simultaneously in a different islet positioned within the same chamber, using Green Downward cADDis sensor (Figure 1d). The [cAMP]_i_ signal was, however, faster to relax to its basal value than the PKA signal, upon removal of forskolin and IBMX (Figure 1d).

The specific agonists, GLP-1 and GIP were applied at three different concentrations, 1 pM, 100 pM and 10 nM, followed by returns to the basal condition (imaging solution supplemented with 10 mM glucose). The agonists induced reversible changes in the YFP/CFP fluorescence ratio (R) (Figure 2a). To identify α-cells within the islets, we then applied 10 μM adrenaline, which is known to elevate [cAMP]_i_ selectively in these cells but, in contrast, inhibit the cAMP signaling in other types of islet cells [32]. The adrenaline response in α-cells has been proved to have a strong correlation with expression of fluorescent markers under a tissue-specific glucagon promoter [5].

### 3.1. Correcting the Time-Dependent Drift of Fluorescence 

The raw traces demonstrated substantial cell-to-cell variation of the YFP/CFP fluorescence ratio, *R*, as well as the presence of a slow time-dependent trend in *R* kinetics (Figure 2a,b): each time the tissue was subject to the basal, agonist-free, conditions, the apparent ratio of fluorescence was decreasing with time. We accounted for the cell-to-cell variation by normalizing *R* of each individual cell to its value at the beginning of the experiment (*R/R*_0_, Figure 2c,d). This correction also increased the signal-to-noise ratio (SNR, Table 1). 

To correct the baseline trend, we performed a subtraction of different baseline functions (Figure S1) from the *R/R*_0_ timecourse. The quality criteria for the baseline correction were (i) the enhancement of the SNR and (ii) the ability to resolve the effects of every compound added. Linear and exponential correction, relying on the first and the last $t_{basal}$ regions (see Methods in Section 2), provided a substantial improvement in the SNR (Table 1) but failed to reveal small changes in the signal, induced by adrenaline (Figure S1a,b). The high-degree polynomial and especially spline correction, utilizing all the $t_{basal}$ regions (“*R*_0_” in Figure S1c,d), had a better capability for resolving the small adrenaline effect (Figure S1c,d). The latter corrections did however introduce several artefacts into the data. The piecewise approach implementing linear (Figure S1e) or square (Figure S1f) fit between each pair of neighboring $t_{basal}$ regions resulted in a significant increase of the SNR and, in the case of the linear correction, excellent resolution of the small adrenaline effect (Figure S1e). We therefore used the piecewise linear correction as a routine throughout the study (Figure 2e,f).

### 3.2. Scaling Up the Unsupervised Quantification of the Effects

Quantification of the (ant)agonist effects (Figure 2) assumes comparing the average *R/R*_0_ values before (red in Figure S2a) and after (blue in Figure S2a) the application of the (ant)agonists. To limit the manual input into the image and data processing to supervision of the ROI detection and entering the timestamps vector, we sought the ways of reusing the $t_{basal}$ regions defined during the baseline subtraction step. To that end, we algorithmically expanded the $t_{basal}$ regions, so they included both the pre- and posteffect signal (Figure 2e), and quantified the (ant)agonist effects either by fitting the signal within each region with different functions (Figure S2b,c,e,f) or as a crude difference between the final and initial fluorescence within each region (Figure S2d). In our hands, the sigmoid and Hill fit provided the best approximation of the (ant)agonist effects (Table 2). Applied to the “real word” data though, the two transcendental fits did not converge in ~2–5% of cells, which prompted us to use a less precise but more stable linear fitting algorithm (Figure S2b) for the quantification of the (ant)agonist effects.

### 3.3. Exploratory Analysis of Cell Populations Based on the Response to Various Stimuli

The incretins GLP-1 and GIP, secreted by the gut L- and K-cells, respectively, are natural peptide factors that target pancreatic islet cells and induce increases in [cAMP]_i_ [18]. In contrast, the catecholamine adrenaline is a body’s soluble signal that has a clear differential effect on two major islet cells subpopulations: it inhibits secretion of insulin by β-cells and induces secretion of glucagon from α-cells to rescue extreme hypoglycemia [32]. The signals induced by incretins and adrenaline are mediated via changes in [cAMP]_i_, acting by increasing or decreasing the concentration of the secondary messenger, respectively. We therefore used adrenaline as a marker compound [17], which discriminates between β- and α-cells within the islet, thereby classifying each cell within the islet solely by its function, without any immunostaining, which would have required killing the cells and permeabilizing the cell membranes (Figure 3). To that end, having used the PKA activity as a surrogate for [cAMP]_i_ (Figure 3a), we have ranked the imaged cells by the change in the PKA activity induced by 10 μm adrenaline (Figure 3b, arrow). Having logically sorted the heterogeneous population into α- (dashed) and β-cell populations (Figure 3b), we observed that mouse α-cells were seemingly better responsive to GLP-1 (and GIP) than β-cells (Figure 3b), in contrast with earlier reports on the limited impact of GLP-1 on [cAMP]_i_ in α-cells [33]. Further ranking of cell responses according to the individual cell sensitivity to GIP or GLP-1 (Figure 3c,d) suggested that the most GLP-1-sensitive cells are at the same time the most GIP-sensitive, within low, physiologically relevant concentrations of the two incretins (1 and 100 pM) (Figure 3c).

### 3.4. Multiparameter Profiling of Cell Subpopulations within Islets

**Figure 4:**
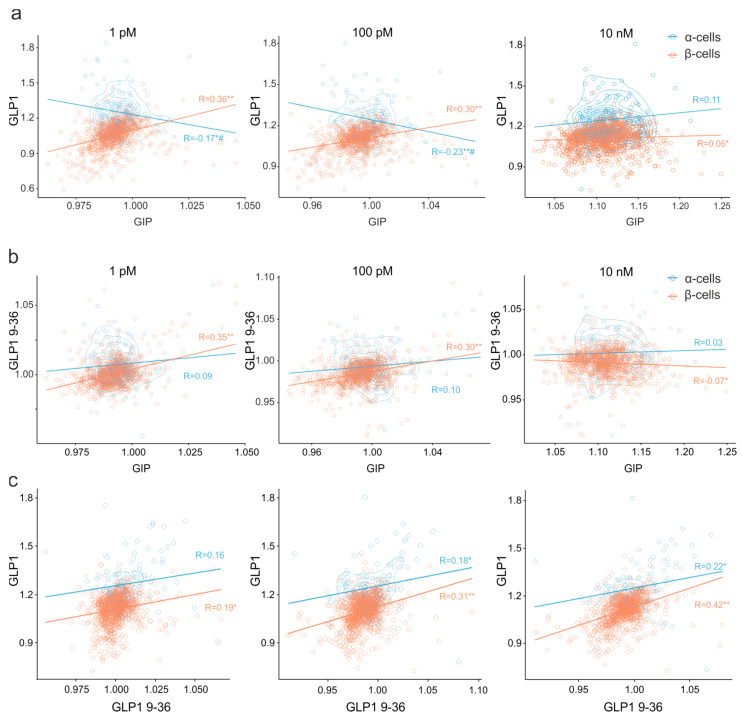
Dose-dependent effects on incretins on α- and β-cell populations in pancreatic islets of Langerhans. 2D scatter plots reporting the effects of incretins, GLP1 7–36 vs. GIP (**a**), GLP1 9–36 vs. GIP (**b**) and GLP1 7–36 vs. GLP-1 9–36 (**c**), added at 1 pM, 100 pM and 10 nM, on α- and β-cells, as indicated. α- and β-cells were distinguished by the positive and negative effect of adrenaline, respectively. The lines represent the least-squares fit, the Pearson’s R-values being given for α- and β-cell subpopulations, as indicated. The data is from three experiments, n = 4096 cells. Correlations are statistically significant at * *p* < 0.05 or ** *p* < 0.01.

We have therefore computed correlations between the effects of the two incretins, GIP and GLP-1, on a per-cell basis, within α- and β-cell populations. Palpable at low and intermediate concentrations (1 pM, 100 pM), positive per-cell correlation between GIP and GLP-1 effects in the β-cell population was significantly decreased when the incretins were used at 10 nM (Figure 4a). A similar pattern was observed for GIP and GLP-1 9–36 (Figure 4b). Overall, despite the low potency of the inactive form of GLP-1 (9–36) (Figure 3d), the effects of the two forms of GLP-1 appeared to associate in statistical sense, in the β-cells (Figure 4c), inclusive of the concentration-dependent decline in correlation with GIP (cf. response to 100 pM and 10 nM among the red markers in Figure 4a,b). For α-cells, however, the correlation between the effects of GLP-1 and GIP, at physiologically relevant concentrations of 1 and 100 pM, was negative, which, just like in the case of β-cells, was attenuated at the higher concentration of the agonists (blue in Figure 4b).

### 3.5. Clusters of Islet Cells Responding to the Incretin Signals 

**Figure 5:**
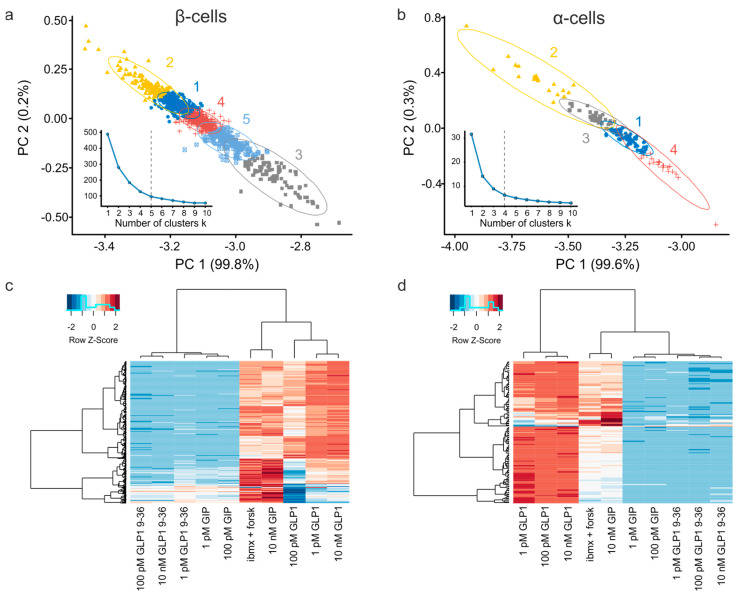
Cluster analysis of the incretin responsiveness of pancreatic islet cells. (**a**,**b**) k-means clustering of β- (**a**) and α-cell (**b**) populations. The linear contributions of individual factors into the first two principal components, PC 1 and PC2, are detailed in Table S2. *Insets:* Total within-cluster sum of squares depending on the cluster number. The plots were used to determine the optimal number of clusters. (**c**,**d**) Hierarchical clustering of pancreatic β- (**c**) and α-cell (**d**) populations alongside the incretin stimuli. “GLP9” denotes the inactive form of GLP-1, GLP-1 9–36. Z-score is the value relationship to the mean of the row, in the units of the standard deviation. Note a close per-cell association between the effects of lower concentrations of GIP (1 pM, 100 pM) and the inactive form of GLP-1 (9–36) as well as the effects of the high concentration of GIP (10 nM) and that of a direct stimulation of adenylate cyclase/inhibition of phosphodiesterase by forskolin and IMBX. The data is from six experiments, n = 10,294 cells.

We further probed the association of the incretin effects in two main islet cell populations, α- and β-cells, by performing the cluster analysis of the functional response to three concentrations (1 pM, 100 pM, 10 nM) of the agonists from six pancreatic islet preparations (n = 10,294 cells). The k-means cluster analysis (Figure 5a,b) allowed mapping of five (for β-cells) and four (α-cells) functionally distinct subpopulations (Figure 5a,b). The major contributors into the principal component 1 (PC 1) were the effects of the three concentrations of GLP-1 whereas PC 2 is mostly influenced by GLP-1 (9–36) (Table S1, Table S2) for both α- and β-cells. 

The hierarchical clustering (Figure 5c,d: dendrogram along the X-axis) revealed a strong association between the responses to the three concentrations of GLP-1 as well as between the responses to all concentrations GLP1 (9–36) in both β- (Figure 5c) and α-cells (Figure 5d). At the same time, the response to GIP displayed nonmonotonous concentration-dependent behavior (Figure 5c,d): 1 and 100 pM clustered with the inactive form of GLP-1 (9–36) whereas the 10 nM data clustered with the effect of IBMX and forskolin, which directly stimulate adenylyl cyclase and inhibit phosphodiesterase.

## 4. Discussion

We report a framework for deep functional profiling of cell subpopulations within the living tissues. The approach, based on real-time imaging of reversible effects imposed by various physiologically relevant (ant)agonists, reflected in changes of the concentration of an intracellular messenger ([cAMP], in our case).

### 4.1. Technical Aspects

#### 4.1.1. Cell Detection

The key weakness of wide-field epifluorescence microscopy is the out-of-focus fluorescence resulting in a poor discrimination of the signal between neighboring cells, which renders the technique unusable for true 3D imaging. Whilst allowing a better cancellation of the out-of-focus light, laser scanning confocal microscopy delivers a lower signal-to-noise ratio [5]. The latter represents a critical issue for imaging of large populations that benefits from the large field-of-view typical for lower magnification objectives that tend to have a lower numerical aperture and hence lower sensitivity. In our case, a compromise solution is a 2D imaging of the cells lying on the periphery of the tissue that stems from the expression pattern of the fluorescent reporter delivered by an adenovirus [5,24]. 

A strong advantage of the genetically encoded sensor is that its fluorescence is strictly contained to the cells thereby limiting the extracellular artefacts and simplifying the detection of cells. In our system where CFP is perfectly colocalized with YFP in the cell, as two domains of the same molecule, we used the brighter YFP channel to map the ROIs. Rather than performing a pixel-by-pixel ratio of YFP and CFP fluorescence intensity use ROI-by-ROI ratio to reduce noise stemming from any small misalignment of the two channels.

#### 4.1.2. Choice of Reporter

The wide variety of the receptor proteins residing on the cell membrane sense a plenitude of extracellular stimuli, which converges into a very limited number of intracellular signals, such as Ca^2+^, diacyl glycerol and cAMP. Of the three signals, [Ca^2+^]_i_ assays have historically seen the greatest progress, due to the triggering nature of this signal and its close association with cell membrane excitability. However, imaging of [Ca^2+^]_i_ is less compatible with large sample sizes and experimental duration due to the demand for a high acquisition rate (ca.10s) needed to resolve calcium dynamics. In this sense, long large-sample-size recordings favor slower, less “regenerative” signals, such as [cAMP]_i_, that can be imaged on a minutes timescale and reflect equally high number of signaling pathways thereby guaranteeing the profiling depth. The obvious down side of this choice is a relatively narrower offer of the recombinant [cAMP]_i_ sensors.

#### 4.1.3. Depth of Profiling

The practical determinant of the depth of the cell profiling in our system (the ability to record the effects of multiple agonists added sequentially to the same cell) is the maximal duration of the experiment. Having minimized the exposure to the exciting light by the choice of the sensor, we attenuate its effect on cell viability and the brightness of the fluorescent sensor. The remaining “real-world” factor limiting the recording duration is the mechanical stability of the imaged object. Whereas small movements can be accounted by the existing image registration routines [34], a rapid or substantial motion cannot be corrected analytically. Our approach to this problem was avoiding the motion by applying a small mechanical pressure on the imaged object [24]. Furthermore, the use of the open-air design [24,35], whereby the solution is blotted through the imaging chamber [24], dampens any pulsations and removes the air bubbles produced by pumping and liquid handling.

#### 4.1.4. Baseline Correction

Oxygen-dependent reduction of the sensor fluorescence (photobleaching) is proportional to the excitation power [36,37], thus minimizing that whilst using a longer exposure times allows monitoring the dynamics of slow (0.5–1 min acquisition intervals) signals reliably for about 2 h, after which the baseline correction becomes vital. The ratiometric nature of the PKA sensor employed in this study does not rescue the baseline drift, as the photobleaching rates of the donor and the acceptor are different [38]. FRET sensors are thus paradoxically more prone to the bleaching-related artefacts [39]. Likewise, the fourfold difference in brightness (the product of the extinction and the quantum yield) of the two fluorophores [40] renders the FRET pair sensitive to factors like pH.

### 4.2. Physiological Significance

#### 4.2.1. Functional Profiling Reflects Cell Heterogeneity

The responses to physiological stimuli subject to variability, even within a nominally homogenous cell population, as has been reported for mouse [24,41] and human [24,42] pancreatic islet cells. The islet secretory function is vastly redundant, which safeguards the control over blood glucose levels. For instance, as little as 2% (Pareto: *x_m_* = 0.4, α = 1), of islets’ second largest population, α-cells, are required to maintain body’s glucagon levels [43]; this figure is ca.20% (Pareto: *x_m_* = 4, α = 1) for less abundant δ-cells [44]. The minimal fraction of the largest islet population, β-cells, is hard to determine as these cells are electrically coupled together and therefore function as a single unit. An increase in [cAMP]_i_ in response to 1 pM GLP-1 is a stimulus that does not propagate between neighboring cells. In our hands, [cAMP]_i_ went up in 65%, with a potent sustained response being observed in only 15% of β-cells (Figure 3b,c). The GLP-1 responsiveness is a major factor of per-cell heterogeneity of β-cells (Figure 5a, Table S2), which echoes the GLP-1 receptor per-cell heterogeneity [45] as well as the variability of the response to the GLP-1 mimetic antidiabetic drugs among human patients [46].

#### 4.2.2. Pharmacology of Cell Populations

In our hands, the responses to low (physiological) doses of GIP clustered with the responses to inactive (9–36) form of GLP-1 (Figure 5a,b), whereas, the responses to high (potentially therapeutic) doses of GIP clustered with that of direct agonists of adenylyl cyclase (forskolin) (Figure 5a,b), for both β- and α-cells. Different concentrations of GIP are therefore likely to act via different routes, possibly via receptors of different affinity expressed by different cell subpopulations. The populational approach to the pharmacological data may therefore shed light on the mechanisms of action of the proven and yet-to-be discovered drugs.

## 5. Conclusions

We present key steps of functional profiling of living cells within biological tissue, inclusive of imaging, image processing and data analysis. The profiling can bring novel insights into yet unstudied aspects of human physiology thereby contributing to more specific pharmacological targeting of cell subpopulations. We expect the approach to benefit from developments in budget imaging technology, based on lab-on-a-chip or organ-on-a-chip configurations. We expect that novel recombinant sensors as well as functional data databases to further enhance the impact of our work.

## Figures and Tables

**Table 1:** Signal-to-noise ratio improvement after data correction procedures.

Procedure	SNR, a.u.	Comment
Raw data	23 ± 5 (n = 1300)	Per-cell variation of fluorescence and baseline drift
Normalized to initial ratio	256 ± 24	Baseline drifts with time
Linear baseline correction	322 ± 44	Ignores small effects
Exp baseline correction	318 ± 40	Ignores small effects
Spline baseline correction	385 ± 15	Introduces artefacts
Poly baseline correction	374 ± 55	Ignores small effects
Piecewise linear baseline correction	410 ± 21	Method of choice
Piecewise square baseline correction	395 ± 28	Introduces artefacts

**Table 2:** Root square means of differences between the agonist effect assessed via the two-region approach (Figure S2a) and single-region algorithms (Figure S2b–f).

Procedure	RMS vs. the Two-Region	Comment
Two-region	0	Bona fide but time-consuming
Linear	0.152 ± 0.053 (n = 1300)	Method of choice
Square	0.841 ± 0.122	Introduces artefacts
End–start	0.363 ± 0.094	Requires smoothing, prone to artefacts
Sigmoid	0.023 ± 0.008	Precise but fitting needs supervision
Hill	0.022 ± 0.007	Precise but fitting requires supervision

## Data Availability

Not applicable.

## Supplementary Materials

The following are available online at https://www.mdpi.com/2313-433X/7/1/9/s1, Figure S1: Baseline correction using different algorithms, Figure S2: Unsupervised quantification of the agonist effects, Table S1: Principal components of the k-means clustering (β-cells) and the contributions of the responses to the peptide agonists, Table S2: Principal components of k-means clustering (α-cells) and the contributions of the responses to the peptide agonists.

## Abbreviations

GLP-1, glucagon-like peptide-1; GLP-1 (9–36), inactive form of glucagon-like peptide-1; YFP, yellow fluorescent protein; DPP-4, dipeptidyl peptidase 4; GIP, glucose-dependent insulinotropic peptide.
