# Supplementary material for: Imaging Meets Cytometry: Analyzing Heterogeneous Functional Microscopic Data from Living Cell Populations
Source: J Imaging. 2021 Jan 13;7(1):9. doi: 10.3390/jimaging7010009 (PMC8321243; doi:10.3390/jimaging7010009)
Supplement: Supplementary file 1 [file jimaging-07-00009-s001.pdf]

Journal of
Imaging

Imaging meets cytometry: analyzing heterogeneous functional microscopic data from living cell populations

Matthew Draper, Mara Willems, Reshwan K. Malahe, Alexander Hamilton and Andrei I Tarasov

Supplementary figures and tables

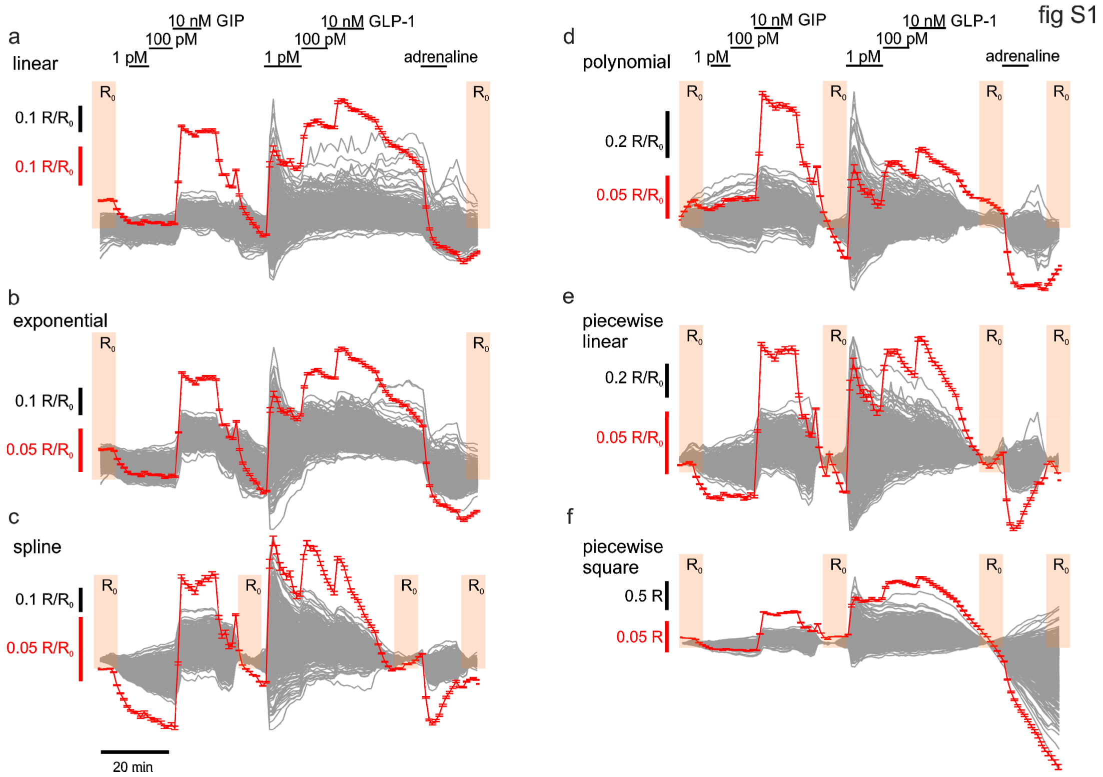

Figure S1. Baseline correction using different algorithms. Whilst baseline correction based on simple linear (**a**) and exponential (**b**) fits resulted in an improvement of the signal-to-noise ratio, both algorithms failed to account for smaller effect like that of adrenaline. This issue was rectified using the spline (**c**) but not polynomial (**d**) correction. However, the spline algorithm failed to de-trend the time-course. Piecewise linear algorithm (**e**) that separated the trace into several regions between the applications of the basal conditions (*R0*) both de-trended the signal and resolved the small effects well. Using a higher degree piecewise fit (**f**), however, introduced several artefacts, just like the polynomial correction did (**d**).

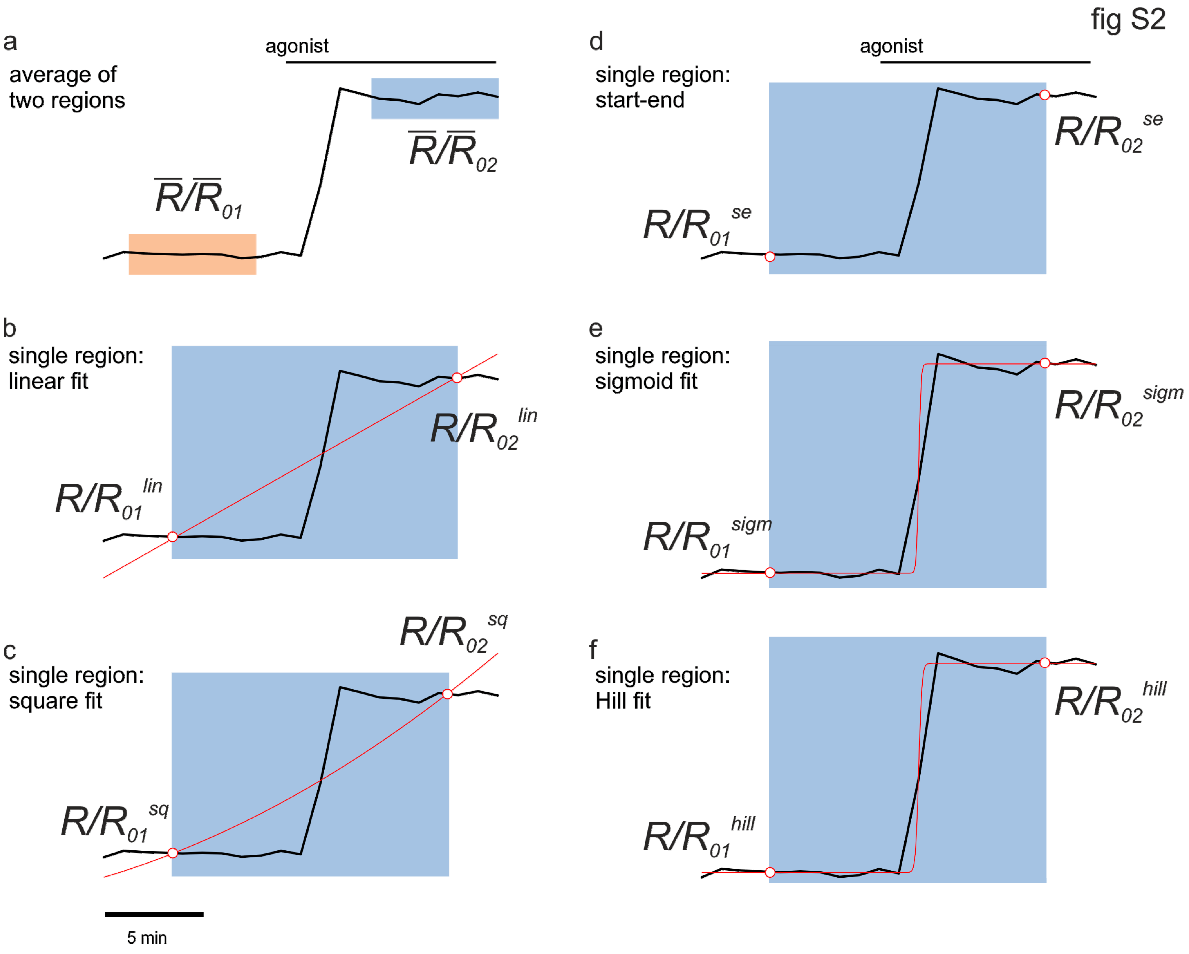

Figure S2. Unsupervised quantification of the agonist effects. (**a**): An ideal approach ("*bona fide*") that takes the means of the two regions of fluorescence, prior and post application of the agonist ($\bar{I}_1$ and $\bar{I}_2$, respectively), for each individual cell, and computes the ratio reflecting the magnitude of the effect. The approach requires a user input step for marking the two regions. (**b**–**f**): Optimization of the quantification procedure for the unsupervised single-region case. A single region containing both pre- and post-agonist signal, with subsequent quantification of the agonist effect based on linear (**b**), square (**c**), sigmoid (**e**), Hill (**f**) fit of the data within the range. The effect is then expressed as the difference in the fitted intensity computed at the points of the beginning and the end of the region. (**d**) simple difference of the fluorescence intensities and the beginning and the end of the region.

Table S1. Principal components of the k-means clustering (β-cells) and the contributions of the responses to the peptide agonists.

	**PC 1**	**PC 2**	**PC 3**	**PC 4**	**PC 5**
1 pM GIP	3.2	13.3	24.9	11.7	1.7
100 pM GIP	2.8	15.3	28.5	1.7	0.1
10 nM GIP	0.2	0.2	16.4	78.9	1.5
1 pM GLP1	23.5	1.8	6.5	2.9	0.6
100 pM GLP1	24.0	3.0	5.5	1.0	0.0
10 nM GLP1	24.0	3.6	4.1	0.7	0.1
1 pM GLP1 9-36	5.0	21.2	4.8	0.4	58.6
100 pM GLP1 9-36	8.4	23.4	4.7	1.0	1.6
10 nM GLP1 9-36	8.9	18.3	4.6	1.7	35.8

Table S2. Principal components of k-means clustering (α-cells) and the contributions of the responses to the peptide agonists.

	**PC 1**	**PC 2**	**PC 3**	**PC 4**	**PC 5**
1 pM GIP	8.9	5.6	25.2	11.4	3.9
100 pM GIP	8.7	2.9	37.6	0.9	0.0
10 nM GIP	0.0	3.3	19.4	69.4	2.2
1 pM GLP1	17.8	11.2	0.4	2.1	1.1
100 pM GLP1	16.2	16.2	0.5	0.0	1.1
10 nM GLP1	14.7	19.0	1.0	0.0	0.4
1 pM GLP1 9-36	6.6	22.1	2.7	0.1	64.5
100 pM GLP1 9-36	12.4	13.8	6.9	7.3	5.7
10 nM GLP1 9-36	14.6	6.0	6.3	8.7	21.1
